# Gastroduodenal Intussusception with Gastric Adenocarcinoma: A Case Report and Review of the Literature

**DOI:** 10.70352/scrj.cr.25-0184

**Published:** 2025-07-01

**Authors:** Ryota Omura, Satoshi Ida, Hiroki Tsubakihara, Keisuke Kosumi, Kazuto Harada, Kojiro Eto, Yuji Miyamoto, Masaaki Iwatsuki

**Affiliations:** Department of Gastroenterological Surgery, Graduate School of Medical Sciences, Kumamoto University, Kumamoto, Kumamoto, Japan

**Keywords:** intussusception, gastric adenocarcinoma, ball valve syndrome

## Abstract

**INTRODUCTION:**

Intussusception in adults is a rare condition, and gastric cancer prolapsing into the duodenum is an even rarer phenomenon. We present a case of early gastric cancer originating in the gastric body with duodenal intussusception and discuss the clinical considerations based on the patient’s overall condition and existing literature.

**CASE PRESENTATION:**

A 69-year-old man with a 30-mm tumor arising from the posterior wall of the gastric body was scheduled for elective surgery. During hospitalization for diabetes mellitus management, he developed sudden epigastric pain and nausea. Upper gastrointestinal endoscopy revealed tumor prolapse into the duodenum, leading to a diagnosis of ball valve syndrome. After successful endoscopic reduction, open local gastrectomy was performed. Pathological examination confirmed a well-differentiated tubular adenocarcinoma, classified as pT1b (SM2) N0M0, pStage IA.

**CONCLUSIONS:**

Gastric cancer with duodenal intussusception is often early-stage and characterized by well-differentiated tubular adenocarcinoma. Depending on the patient’s condition, endoscopic resection or limited surgical resection may be viable treatment options for this rare condition.

## Abbreviation

CT
: computed tomography

## INTRODUCTION

Intussusception in adults is a rare condition, and gastric cancer prolapsing into the duodenum is even rarer.^1)^ Notably, previous reports indicate that such cases are predominantly associated with early-stage gastric cancer.^2)^ While the standard treatment for gastric cancer typically involves gastrectomy, the unique presentation of gastric cancer with duodenal intussusception often raises the possibility of alternative approaches. For patients with significant comorbidities and an elevated risk of postoperative complications, localized resection may serve as a viable option, offering a less invasive yet effective treatment. We herein present a case of early gastric cancer originating in the gastric body with duodenal intussusception and discuss the clinical considerations for selecting limited surgical resection based on the patient’s overall condition and existing literature.

## CASE PRESENTATION

**Fig. 1:**
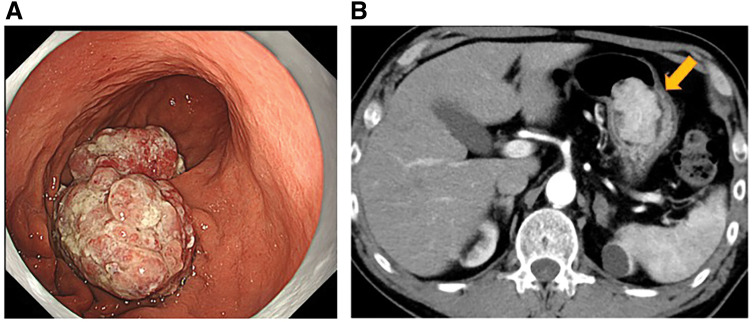
Preoperative findings. (**A**) Upper gastrointestinal endoscopy showed the lesions extended beyond the submucosal layer. (**B**) Contrast-enhanced CT scan showed a 30-mm tumor in the gastric body. The yellow arrow indicates the tumor.

**Fig. 2:**
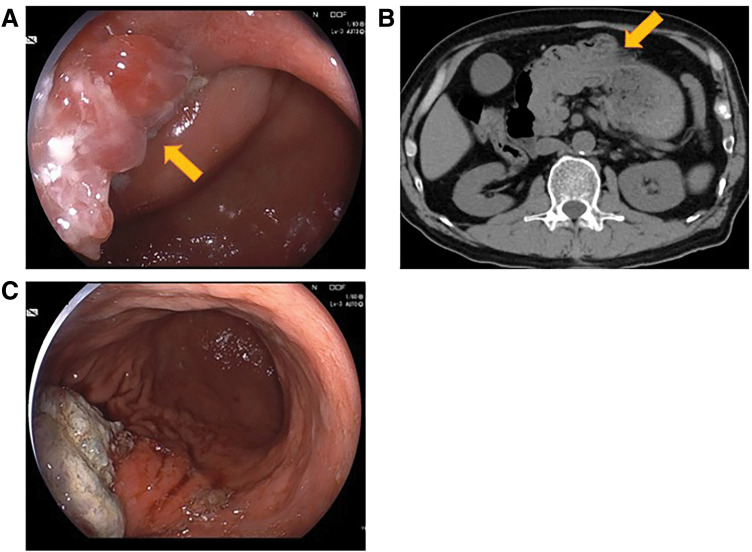
Findings during re-examination after the onset of epigastric pain. (**A**) Upper gastrointestinal endoscopy showed the tumor was shifted toward the duodenum. No additional findings, such as gastrointestinal bleeding, were observed. (**B**) Plain CT scan showed that the tumor had shifted toward the duodenum. (**C**) Upper gastrointestinal endoscopy after the tumor was repositioned into the stomach. The yellow arrow indicates the tumor prolapsing through the pyloric ring.

The patient was a 69-year-old man. Upper gastrointestinal endoscopy at a local clinic revealed a 30-mm, type 0-I, elevated lesion on the posterior wall of the greater curvature of the gastric body (**Fig. 1**). Biopsy results confirmed well-differentiated tubular adenocarcinoma. His medical history included chronic progressive pulmonary aspergillosis, myocardial infarction, stroke, and diabetes. Contrast-enhanced CT showed no evidence of lymphadenopathy or distant metastasis. Based on these findings, gastric cancer (M, Gre, Type 0-I, tub1, cT1bN0M0, cStage I) was diagnosed, and distal gastrectomy was planned. However, during hospitalization, the patient suddenly developed epigastric pain and nausea. In view of the sudden epigastric pain, we considered it necessary to rule out myocardial infarction; therefore, an electrocardiogram and cardiac troponin levels were checked. No abnormalities were found on these tests. CT was performed under the suspicion of acute abdomen. Abdominal CT revealed retained gastric contents, and the tumor had shifted toward the duodenum (**Fig. 2**). Upper gastrointestinal endoscopy showed a narrowing resembling torsion from the lower body of the stomach to the duodenal side, with the tumor located in the duodenum. Endoscopic manipulation successfully repositioned the tumor back into the stomach, and the patient’s symptoms resolved thereafter.

Endoscopic treatment was not considered suitable due to the tumor’s size and depth of invasion. While the standard approach would be laparoscopic distal gastrectomy, the patient’s high surgical risk—due to multiple comorbidities and a preoperative COVID-19 infection—necessitated reconsideration. However, given the tumor’s high mobility, the likelihood of muscular layer invasion was deemed extremely low despite its large size, leading to a diagnosis of early gastric cancer. Therefore, a less invasive surgical approach was considered, and open local gastrectomy was selected as the treatment.

**Fig. 3:**
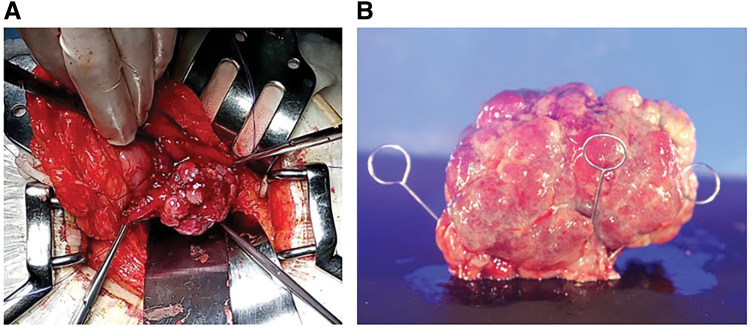
(**A**) Intraoperative findings. The stomach lumen was revealed, exposing the tumor. (**B**) Macroscopic findings revealed a pedunculated lesion measuring 5.5 × 3.7 × 3.5 cm, protruding externally.

Intraoperative findings revealed a palpable mass on the posterior wall of the stomach (**Fig. 3**). Local resection was performed under intraoperative endoscopic guidance, and the gastric lumen was released and resected while checking adequate tumor margins. Additionally, 2 No. 4 lymph nodes, which were visibly enlarged and located near the tumor, were sampled and confirmed the absence of malignancy through intraoperative rapid pathological examination. The operative time was 102 min and estimated blood loss was minimal. In the pathology results, the tumor was diagnosed as a well-differentiated tubular adenocarcinoma with submucosal invasion. The pathological diagnosis was M, Gre, Type 0-I, pT1b2 (SM2), pN0, pStage IA. The surgical resection margins were 7 mm.

The postoperative course was uneventful, and the patient was discharged on postoperative day 15. At 6 months postoperatively, no signs of recurrence were observed.

## DISCUSSION

We encountered a case of gastric cancer that had invaginated into the duodenum. For duodenal intussusception caused by gastric cancer to occur, the tumor must prolapse downward, which is influenced by several factors. First, the tumor must be large enough to enter the duodenum.^1)^ Second, tumor location on a mobile gastric wall, such as the anterior or greater curvature side, is crucial, as opposed to the posterior wall, which is less mobile because of retroperitoneal fixation. Third, the tumor must be an early-stage gastric cancer with minimal infiltration into surrounding structures, preserving its mobility. These factors can lead to the prolapse of a gastric tumor into the duodenum, resulting in occlusion of the pyloric ring and subsequent symptoms such as abdominal pain, vomiting, and abdominal distension, a condition known as “ball valve syndrome.”^3)^

A PubMed search using the keywords “duodenal intussusception” and “gastric cancer” identified 98 articles (cited October 18, 2024). After excluding cases involving benign or submucosal tumors (SMTs) (most of which were gastrointestinal stromal tumors), 9 relevant cases remained. Combined with our current case, a total of 10 cases were analyzed (**Table 1**).^4–12)^ Key characteristics of these cases included a relatively large mean tumor size of approximately 6.5 cm; a predominant tumor location in the lower gastric body, particularly along the greater curvature or anterior wall; and limited depth of invasion, with most tumors confined to the submucosa except for 1 advanced case. This advanced case was treated with adjuvant chemotherapy (a combination of S-1 and docetaxel) for 1 year after surgery according to the gastric cancer guidelines,^13)^ and no recurrence was observed.

**Table 1:** Clinical characteristics of reported cases of gastric cancer with gastroduodenal intussusception

Author	Year	Age	Sex	Symptoms	Tumor location	Tumor size (cm)	Tumor depth	Histologic type	Stage	Treatment	Postoperative observation (month)
Asai et al.	2008	67	M	Nausea	L/Ant	4.5	m	Adenocarcinoma	IA	DG	ND
Eom et al.	2011	73	F	Nausea	L/Post	7.8	mp	Signet ring cell carcinoma	IIA	DG	12
Hirose et al.	2012	70	F	Nausea	L/Gre	7	sm	Adenocarcinoma	IA	DG	ND
Chen et al.	2013	63	F	None	M/Gre	ND	ND	Intramucosal carcinoma	ND	ESD	6
Miyake et al.	2019	84	F	Anemia	L/Gre	6	m	Adenocarcinoma	IA	ESD	ND
Suda et al.	2019	81	F	Abdominal pain	L/Ant	5.5	sm	Adenocarcinoma	IA	LDG	7
Ako et al.	2022	29	F	Abdominal pain	L/Ant	5	sm	Adenocarcinoma	IA	EMR	ND
Namikawa et al.	2023	93	F	Anemia	L/Gre	11.2	sm	Adenocarcinoma	IA	LDG	3
Al-Shamali et al.	2024	75	F	Abdominal pain	L/Gre	11	sm	Adenocarcinoma	IA	DG	3
Present case	2024	69	M	Abdominal pain	M/Gre	5.5	sm	Adenocarcinoma	IA	LR	6

Ant, anterior; DG, distal gastrectomy; EMR, endoscopic mucosal resection; ESD, endoscopic submucosal dissection; F, female; Gre, greater curvature; L, lower 3rd of the stomach; LDG, laparoscopic distal gastrectomy; LR, local resection; m, mucosa; M (in Sex column), male; M, middle 3rd of the stomach; mp, muscularis propria; ND, not described; post, posterior; sm, submucosa; Stage, clinical stage in accordance with the 8th International Union Against Cancer (UICC) Tumor-Node-Metastasis classification

The patient’s symptoms of abdominal pain and vomiting were likely caused by increased gastric peristalsis during food intake, leading to the tumor’s invagination into the duodenum. This hypothesis aligns with the clinical course because symptoms recurred after resuming oral intake following endoscopic reduction of the tumor’s invagination, whereas fasting management effectively prevented recurrence.

Regarding treatment, prompt reduction of the invagination and appropriate surgical resection are recommended. In this case, the prolapse was successfully reduced endoscopically, allowing for an elective gastrectomy. However, because of multiple systemic comorbidities, the American Society of Anesthesiologists physical status was 3, and the predicted 30-day postoperative mortality rate was 3.5% according to the National Clinical Database risk calculator,^14)^ indicating a high-risk patient. Therefore, instead of distal gastrectomy, the standard treatment, we opted for local gastric resection. The rationale for this decision was that, as mentioned above, tumors prone to prolapse are generally early-stage gastric cancers with a low likelihood of lymph node metastasis. Instead, to ensure complete tumor resection while minimizing gastric resection, we performed an open local gastric resection with intraoperative endoscopic assistance. Although we initially considered laparoscopic endoscopic cooperative surgery, the tumor exceeded 5 cm in size and, unlike SMTs, required an appropriate surgical margin because it was gastric cancer. As a result, a large gastric defect was anticipated, making endoscopic resection and laparoscopic closure technically challenging. Furthermore, the tumor had a highly elevated morphology, rendering endoscopic submucosal dissection (ESD) extremely difficult. Therefore, we selected an open approach to shorten the operative time and ensure a safe and reliable resection. It has been suggested that tumor exposure in surgery may increase the risk of peritoneal seeding.^15)^ On the other hand, there have been reports of perforation during ESD for early gastric cancer without subsequent peritoneal dissemination due to perforation.^16)^ Therefore, considering the risk of peritoneal seeding due to exposure of the tumor, the gastric lumen was opened and resected while carefully confirming the tumor margins. Considering the risk of peritoneal dissemination due to tumor exposure, the gastric wall was grasped with forceps to prevent spillage of gastric contents into the abdominal cavity. At the end of the procedure, thorough peritoneal lavage was performed. This case highlights that for high-risk patients, such as the one presented here, less invasive surgical options may be a viable alternative.

## CONCLUSIONS

We have presented a case of gastric body cancer that prolapsed into the duodenum and was managed with endoscopic repositioning followed by surgical resection. In cases of early gastric cancer presenting with elevated lesions, the sudden onset of gastrointestinal symptoms such as abdominal pain and vomiting should raise suspicion for this condition. Because these tumors are typically early-stage gastric cancers, less invasive surgical options may be a viable choice for high-risk patients.

## DECLARATIONS

### Funding

None of the authors received any funding for this study.

### Authors’ contributions

RO drafted the manuscript.

RO, SI, HT, KK, KH, KE, YM, and MI determined the treatment plan.

RO, SI, and MI performed the surgery.

All authors read and approved the final manuscript.

### Availability of data and materials

The data and materials used in this case report can be made available upon reasonable request to the corresponding author, within the scope of the patient’s consent.

### Ethics approval and consent to participate

This work does not require ethical considerations or approval. Informed consent to participate in this study was obtained from the patient.

### Consent for publication

Written informed consent for publication was obtained from the patient.

### Competing interests

The authors have no conflicts of interest to report.
